# p53 Increases Intra-Cellular Calcium Release by Transcriptional Regulation of Calcium Channel TRPC6 in GaQ_3_-Treated Cancer Cells

**DOI:** 10.1371/journal.pone.0071016

**Published:** 2013-08-16

**Authors:** Esha Madan, Rajan Gogna, Bernhard Keppler, Uttam Pati

**Affiliations:** 1 Transcription and Human Biology Laboratory, School of Biotechnology, Jawaharlal Nehru University, New Delhi, Delhi, India; 2 Institute of Inorganic Chemistry, Vienna University, Vienna, Austria.; Virginia Commonwealth University, United States of America

## Abstract

p53 and calcium signaling are inter-dependent and are known to show both synergistic and antagonistic effects on each other in the cellular environment. However, no molecular mechanism or cellular pathway is known which shows direct regulation between these important cellular signaling molecules. Here we have shown that in cancer cells treated with anti-neoplastic drug GaQ_3_, p53, there is an increase in intracellular calcium levels by transcriptional regulation of a novel calcium channel gene TRPC6. p53 directly binds to a 22 bp response element in the TRPC6 gene promoter and increase its mRNA and protein expression. Over-expression of TRPC6 results in calcium-dependent apoptotic death and activation of apoptotic genes in a variety of cancer cells. This research work shows that p53 and its transcriptional activity is critical in regulation of calcium signaling and an increase in the intracellular calcium level might be one of the anti-cancer strategies to induce apoptosis in cancer cells.

## Introduction

Gallium and its organic derivatives have shown high consistency and efficiency as anti-cancer drugs [1]–[5]. We have recently established a novel organic derivative of gallium “GaQ_3_” [tris(8-quinolinolato)gallium(III)] (KP46) as an effective anti-cancer drug in cancer cells with Wt-p53 or Mt-p53 protein [6]. We observed that GaQ_3_ induces calcium signaling in cancer cells by increasing the intracellular calcium levels. Increase in cellular Ca^2+^ activates p53 protein and increases p53 cellular levels. GaQ_3_-induced intracellular calcium release was significantly higher in cancer cells with wild-type p53 than in cancer cells with mutant p53 or with p53 gene deletion [6]. Interestingly, it was observed that the rise in intracellular Ca^2+^ release was p53-dependent and inhibition of p53 transcriptional activity using pifithrin-α abolished the intracellular Ca^2+^ release. This observation suggested that p53 might transcriptionally regulate intracellular Ca^2+^ release and Ca^2+^ signaling in GaQ_3_-treated cancer cells. p53 and calcium are known to function in synergy, but no direct relation has been established between p53 activation and p53-dependent regulation of calcium signaling at the cellular, biochemical or molecular level. In certain reports Ca^2+^-induced signals like Ca^2+^-activated RAF/MEK/ERK pathways mediated p53-independent apoptosis [7]. It is also predicted that p53 works in close relation with cellular calcium signaling, since intracellular calcium release plays an important role in inducing Bcl-2, ROS and mitochondrial pathway of apoptosis [8]. However, no molecular mechanism or pathway of p53-medited regulation of intracellular calcium release is known.

In this study we have shown that the cellular calcium signaling and intracellular calcium release are under transcriptional control of p53 protein. p53 transcriptionally regulates a novel calcium channel TRPC6 by directly binding to a 22 base-pair p53-RE present 400 base pairs upstream of the +1 transcriptional start site (TSS) at the TRPC6 promoter. We observed that GaQ_3_ induces apoptosis via p53-dependent upregulation of TRPC6 gene in cancer cells with Wt p53. Over-expression of TRPC6 results in significant apoptosis in cancer cells. Further TRPC6 expression initiates a calcium-dependent regulation of the expression of genes involved in apoptosis.

## Materials and Methods

### Cell culture

MCF-7, U2OS, HCT, A-431, PC3 and H1299 cells were obtained from National Centre for Cell Science, Pune (India) and were maintained in DMEM medium. The cells were cultured as monolayers in DMEM medium supplemented with 10% (v/v) heat-inactivated fetal bovine serum and antibiotics, and incubated at 37°C in a humidified atmosphere of 95% air and 5% CO_2_ All the transfections were carried out using effectene transfection reagent (qiagen) according to manufacturer's instructions. For the time course analysis 5 petri dishes for each time point were used.

### Plasmids and reagents

p53-TRPC6 full length promoter, p53-TRPC6 minimal promoter and p53-TRPC6 mutant minimal promoter were cloned in pGL3 luciferase vector. p53 si-RNA was also used as described previously by [9]–[11].

### Assay for Ca^2+^mobilization

Ca^2+^ was measured using the cell permeable Ca^2+^ sensitive fluorescent dye Fluo- 3 acetoxymethyl ester. Where indicated, BAPTA acetoxymethyl ester (10 µM) was added to the culture medium of cells in 10-cm plastic tissue culture plates for a 1-h exposure prior to the loading procedure with Fluo-3 acetoxymethyl ester. The medium was removed from the tissue culture plates and replaced with 4 µM Fluo-3 acetoxymethyl ester diluted in Krebs-Ringer buffer (KRB) (10 mM D-glucose, 120 mM NaCl, 4.5 mM KCl, 0.7 mM Na2HPO4, 1.5 mM NaH2PO4, and 0.5 mM MgCl2 (pH 7.4 at 37°C)) (Sigma) for 20 min. The dishes were washed once with 5 ml KRB to remove the residual dye. The cells were harvested by trypsinization, washed in 5 ml of Ca^2+^ free PBS at 37°C, pelleted by centrifugation, re-suspended in 1 ml of Ca^2+^ free PBS at 37°C, and analyzed immediately for Fluo-3 fluorescence intensity by flow-cytometry.

Please refer to File S1 for full description of material and methods.

## Results

### GaQ_3_ induces TRPC6 gene expression in cancer cells with wild-type p53

**Figure 1:**
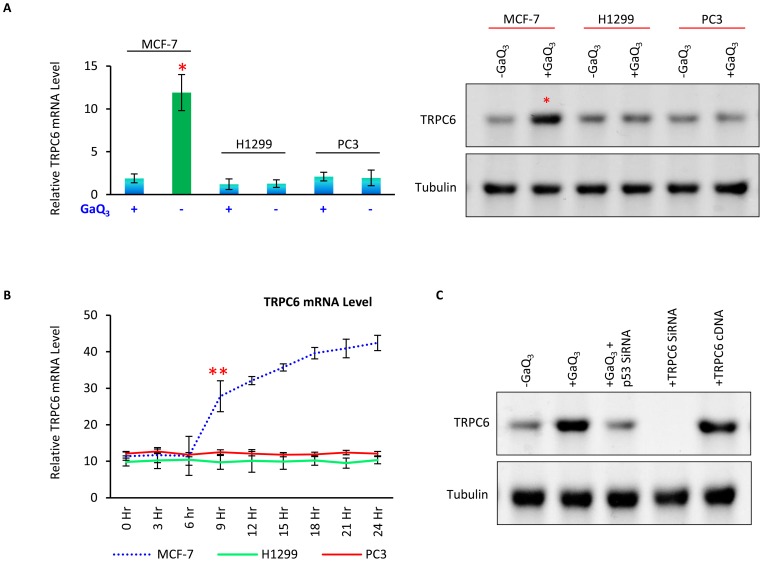
GaQ_3_-induces TRPC6 in cancer cells with WT p53. **A**) The effect of GaQ_3_ upon the expression of TRPC6 mRNA in p53 (+/+) MCF-7, p53 (−/−) H1299 and p53 (mt/mt) PC3 cells is observed using qPCR. Results show that GaQ_3_ can induce up-regulation of TRPC6 only in MCF-7 cells with Wt p53 (lane 2). Similarly GaQ_3_ can induce the expression of TRPC6 protein only in MCF-7 cells (n = 5). **B**) Real-time PCR shows the time course analysis of TRPC6 mRNA expression in GaQ3-treated MCF-7 (black line), H1299 (green line) and PC3 (red line) cells. Results show that TRPC6 mRNA is upregulated only in MCF-7 cells and not in H1299 and PC3 cells. Further, the rise in TRPC6 mRNA is observed at the 6^th^ hr of GaQ3 incubation. The rise in TRPC6 mRNA is temporally related with the rise in p53 mRNA in GaQ3-treated MCF-7 cells (*(red) represents significant difference in the results between red, green and black line at the 9^th^ hr time point, p<0.028), n = 5, Anova, error bars represent S.D). **C**) The role of p53 in GaQ_3_-induced upregulation of TRPC6 is observed. p53 gene silencing using p53 siRNA in GaQ_3_-treated cells reverse the increase in TRPC6 protein level (lane 3) (*(red) represents significant difference in the results between lane 1 & 2, p<0.036 and lane 2 & 3, p<0.042). The efficiency of TRPC6 siRNA and TRPC6 cDNA are shown in lane 4 and 5.

We had earlier observed that GaQ_3_ induces high intracellular calcium release selectively in cancer cells with wild-type p53 protein [6]. Silencing of p53 gene using p53 SiRNA and inhibition of p53 transcriptional activity using pifithrin-α both abolished the GaQ_3_-induced rise in the intracellular calcium release [6]. This data suggested that intracellular rise of calcium levels in cancer cells was regulated by p53 and was dependent on p53 transcriptional activity. However the mechanism involved in the regulation of this observed phenomenon is unknown [6]. Since GaQ_3_-induced significant increase in calcium uptake, using qPCR we analyzed the expression of a large number of known calcium channels in GaQ_3_-treated MCF-7 cells. We observed significantly high expression of TRPC6 gene in GaQ_3_-treated cells. Since the expression of TRPC6 calcium channel was high, we asked if TRPC6 might be involved in the increase in the cellular calcium levels in GaQ_3_-treated cancer cells. The expression of TRPC6 mRNA and protein was observed in GaQ_3_-treated MCF-7, H1299 and PC3 cells **(** **Figure 1** **A)**. The qPCR analysis shows that TRPC6 mRNA was significantly increased in GaQ_3_-treated MCF-7 (p53 Wt) cells **(** **Figure 1A** **)**. The increase in the p53 mRNA level was not observed in H1299 (p53 null) and PC3 (p53 mut) cells **(Bars 3–6;** **Figure 1A** **)**. Similarly p53 protein level was analyzed in the above mentioned cancer cells and only MCF–7 showed significant increase in p53 protein level upon GaQ_3_ treatment **(** **Figure 1A** **)**. This data suggested that p53 played important role in GaQ_3_-mediated upregulation of TRPC6. Further the relation between the p53 expression and the TRPC6 expression is observed. The time-course analysis of TRPC6 mRNA level (real-time PCR) in GaQ_3_-treated MCF-7, H1299 and PC3 cells show that TRPC6 mRNA was significantly high in p53 (+/+) MCF-7 cells **(** **Figure 1B** **; black line)**. Time-course analysis shows that GaQ_3_ induces the expression of TRPC6 within 6 hr of its incubation and the TRPC6 expression increases from 6^th^ to 24^th^ hr in a similar pattern in which p53 expression increase in GaQ_3_-treated cells [6] ref is now placed. This data suggests that p53 and TRPC6 show temporal relation in their mRNA expression profiling. The direct role of p53 in the expression of TRPC6 in GaQ_3_-treated MCF-7 cells is observed by silencing p53 gene in GaQ_3_-treated MCF-7 cells **(** **Figure 1C** **)**. The results show that TRPC6 protein expression was significantly high upon GaQ_3_ treatment (lane 2); however this GaQ_3_-induced rise in TRPC6 was completely reversed upon p53 silencing (lane 3). This data suggests that p53 has an important role in the regulation of TRPC6 gene. We further analyzed the efficiency of TRPC6 siRNA and TRPC6 cDNA (2 μM) in MCF-7 cells.

### p53 transcriptionally activates TRPC6 promoter

**Figure 2:**
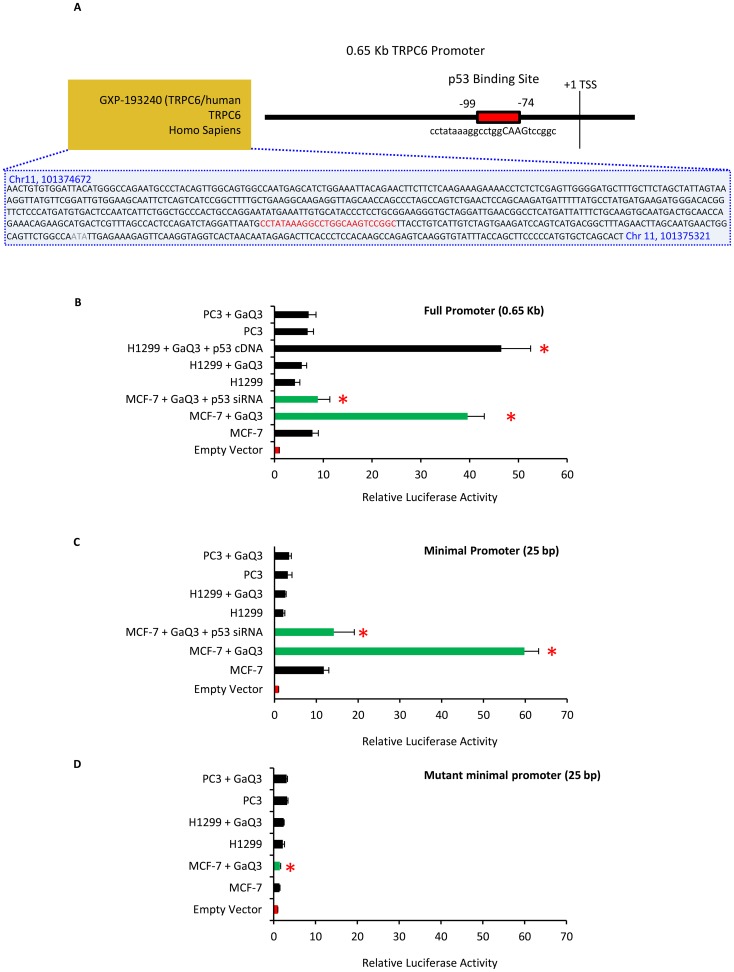
p53 transcriptionally regulates TRPC6 promoter. **A**) A putative p53 binding site is observed in the TRPC6 promoter using Genomatix, Matinspector module. TRPC6-p53RE lies between −422 to −400 bp on the 0.6 kb TRPC6 promoter. The DNA seq of the TRPC6 promoter along with the location of p53 RE (shown in red) is diagrammatically represented. **B**) pTRPC6p-luc1 (TRPC6 0.6 kb promoter luciferase construct) is transfected in GaQ_3_-treated MCF-7 cells and effect of p53 on luciferase activity is measured. GaQ_3_-induced p53 gene activation induces 8-fold increase in pTRPC6p-luc1 luciferase activity (bar 3). p53 gene silencing using p53 siRNA reverses this effect and TRPC6 promoter activation is abolished (bar 4). Transfection of pTRPC6p-luc1 in H1299 and PC3 cells in absence or presence of GaQ_3_ has no effect on the activity of TRPC6 promoter. Upon exogenous addition of Wt p53 cDNA in GaQ_3_-treated H1299 cells the TRPC6 promoter shows 9-fold increase in the luciferase activity (bar 7). This data show that p53 regulates TRPC6 promoter transcriptionally. (**C**) pTRPC63p-luc2, the TRPC6-p53RE (−422 to −400) cloned in luciferase vector is transfected in GaQ_3_-treated MCF-7 cells. Results show that p53 induces a 6-fold increase in the TRPC6-p53RE luciferase activity (bar 3). p53 gene silencing using p53 siRNA abolishes the increase in luciferase activity (bar 4). The increase in TRPC6 promoter activity is absent in H1299 and PC3 cells. The data show that p53 regulates TRPC6 promoter via TRPC6-p53RE. (**D**) The mmpTRPC6p-luc2 construct with mutated sequence of TRPC6-p53RE is transfected in GaQ_3_-treated MCF-7 cells. No increase in the luciferase activity is observed, showing the specificity of TRPC6-p53RE. For figure 2b–2d, (*(red) represents significant difference in the results all values p<0.05), n = 7, Anova, error bars represent S.D).

TRPC6 (chromosome 11q22.1, reverse strand) and p53 showed temporal relations in their expression profile in GaQ_3_-treated cancer cells, therefore it was of interest to define the role of p53 in regulation of TRPC6. The TRPC6 promoter region was identified as 650 bp DNA sequence upstream of +1 transcription start site, using matrix matches determined by Mat Inspector (Genomatix). TRPC6 promoter was provided the locus ID GXP_193240 by the Mat Inspector. This region lies between regions 101,374,672–101,375,321 (TSS ref point represented by Mat Inspector is 101,374,772) and is represented by ENST00000527240 and AK027769. Bioinformatics analysis of the TRPC6 promoter using Mat Inspector module of genomatix database showed putative p53 DNA binding site (matrix sim; score>0.9) **(** **Figure 2A** **)** suggesting that p53 might be a potential TRPC6 regulator. To establish this, we cloned a 0.65 kb putative TRPC6 promoter carrying the p53 response element (p53RE) into a pGL3 basic vector to generate pTRPC6p-luc1. The pTRPC6p-luc1 was transfected in untreated and GaQ_3_-treated MCF-7, H1299 and PC3 cells. GaQ_3_ treatment induced 5-fold increase in TRPC6 promoter activity in MCF-7 cells in comparison to the untreated cells **(** **Figure 2B** **; bar 2 and 3)**. The increase in TRPC6 promoter activity was p53-dependent since p53 gene silencing using p53 siRNA reversed the GaQ_3_-induced increase in TRPC6 promoter activity. GaQ_3_ treatment was unable to induce TRPC6 promoter activity in H1299 and PC3 cells (bar 5, 6, 8 & 9). TRPC6 promoter was activated in GaQ_3_-treated H1299 cells which were transfected with p53 cDNA (bar 7). This data showed that TRPC6 was regulated by p53 in GaQ_3_-treated cells. To further confirm the role of p53RE in p53-mediated regulation of TRPC6 promoter, the region between base pairs −74 to −99 (with reference to the TSS seq (101,374,772) of the TRPC6 promoter carrying the p53RE were cloned into a pGL3 vector to generate the minimal pTRPC6p-luc2. This TRPC6 minimal promoter was induced upon GaQ3-treatment in MCF-7 cells and p53 silencing abolished this increase in the promoter activity **(** **Figure 2C** **, bar 2–4)**. In order to establish the role of the newly identified p53RE in p53-mediated regulation of TRPC6 gene, the p53RE sequence was mutated and cloned in PGL3 vector to generate the mutant minimal mmpTRPC63p-luc2. Transfection of mmpTRPC63p-luc2 in GaQ_3_-treated MCF-7, PC3 and H1299 cells showed no increase in the TRPC6 promoter activity **(** **Figure 2D** **)**. This data establishes that p53 transcriptionally regulates TRPC6 in GaQ_3_-treated cancer cells.

### WT p53 directly binds to its response element at the TRPC6 promoter

**Figure 3:**
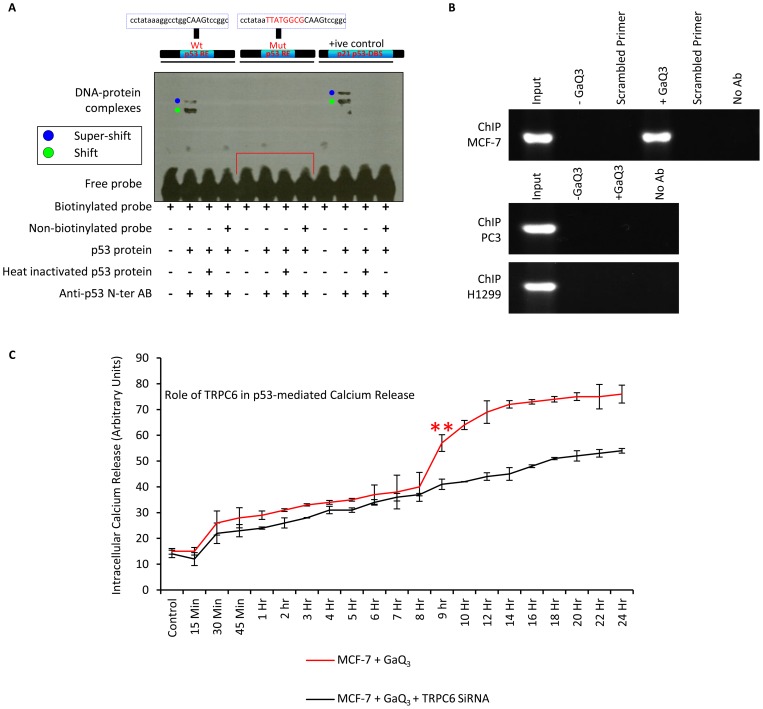
p53 directly binds to the TRPC6 promoter. **A**) EMSA is conducted to study the binding between p53 and its response element at the TRPC6 promoter under *in-vitro* conditions. A shift and a super-shift of the p53, p53AB and the p53-RE are observed in lane 2. The binding between p53 and its RE is lost after a mutant p53 RE sequence is used for EMSA, showing high specificity of this interaction (lane 6), Binding between p53 and its known p215′RE is used as a control (lane 9–12). (**B**) Chromatin immunoprecipitation is conducted in GaQ_3_-treated MCF-7 cells to confirm *in-vivo* binding of p53 to p53RE at the TRPC6 promoter. p53 shows positive TRPC6-p53RE binding exclusively in GaQ_3_-treated cells. Input serves as positive control and scrambled primers for PCR were used as negative control (n = 5). (**C**) The binding between TRPC6 and p53 RE is not observed in H1299 and PC3 cells even in the presence of GaQ_3_ (n = 5). (**D**) Time course analysis of intracellular calcium release is observed in the GaQ_3_-treated MCF-7 cells (red line) and GaQ_3_-treated MCF-7 cells where TRPC6 gene is silenced using TRPC6 siRNA (black line). Results show that GaQ_3_ induces the sharp increase in the intracellular calcium release in p53 (+/+) MCF-7 cells by 6^th^ hr of its incubation. The silencing of the TRPC6 abolishes this 6^th^ hr increase in the intracellular calcium release (*(red) represents significant difference in the results between red and black line at the 8^th^ hr time point, p<0.029), n = 5, Anova, error bars represent S.D).

The binding of p53 at the 22 base pair region at the TRPC6 promoter was analyzed under *in-vitro* conditions using EMSA **(** **Figure 3A** **)**. The data showed binding of p53 at the TRPC6-p53-RE sequence as a clear shift and super-shift of the complex was observed (lane 2). The binding between p53 and its response element was lost upon heat denaturing p53 protein (lane 3). The binding between p53 and its response element was very specific since mutation of the 22 base pair p53 RE sequence abolished the binding between p53 and its RE (lane 5–8). The binding between p53 and its response element at the p21 5′ promoter element serves as positive control (lane 9–12). To further define the role of TRPC6-p53RE in p53-mediated TRPC6 induction under *in-vivo* conditions, we performed chromatin immunoprecipitation (ChIP) assays in GaQ_3_-treated MCF-7 cells. Consistent with luciferase results, we detected one specific PCR product derived from TRPC6-p53RE **(** **Figure 3B** **, upper panel)**. Input serves as the control, in absence of GaQ_3_ treatment p53 showed no binding to the p53RE at the TRPC6 promoter. Upon treatment of MCF-7 cells with GaQ_3_, p53 shows binding to its RE at the TRPC6 promoter, PCR with scrambled primers serves as the negative control. On the other hand no binding between p53 and p53RE at the TRPC6 promoter is observed in H1299 and PC3 cells **(** **Figure 3B** **, lower panel)**. These results established that that TRPC6-p53RE was responsible for p53-mediated induction of TRPC6 promoter activity and that p53 transcriptionally induces TRPC6 through promoter binding in GaQ_3_-treated cells. Since GaQ_3_ induces an exponential increase in the intracellular calcium release in cancer cells with Wt p53 and p53 is now shown to transcriptionally regulate the calcium channel TRPC6 in the GaQ_3_-treated cells, the role of TRPC6 in the p53-mediated calcium release is observed. Flow-cytometric analysis of the intracellular calcium release shows that GaQ_3_ treatment induces an exponential increase in the release of intracellular calcium at the 8^th^ hr of its incubation **(** **Figure 3D** **, red line)**, upon silencing TRPC6 gene using TRPC6 siRNA we observed that the 8 hr exponential increase in the intracellular calcium levels was reversed and the increase in calcium levels became linear as previously observed in p53 null (H1299) and p53 mutant (PC3) cells. This data shows that the GaQ_3_-induced and p53-mediated rise in the intracellular calcium release is due to the p53-dependent transcriptional up-regulation of TRPC6 protein in the treated cancer cells.

### TRPC6 induces calcium-dependent apoptosis in cancer cells

**Figure 4:**
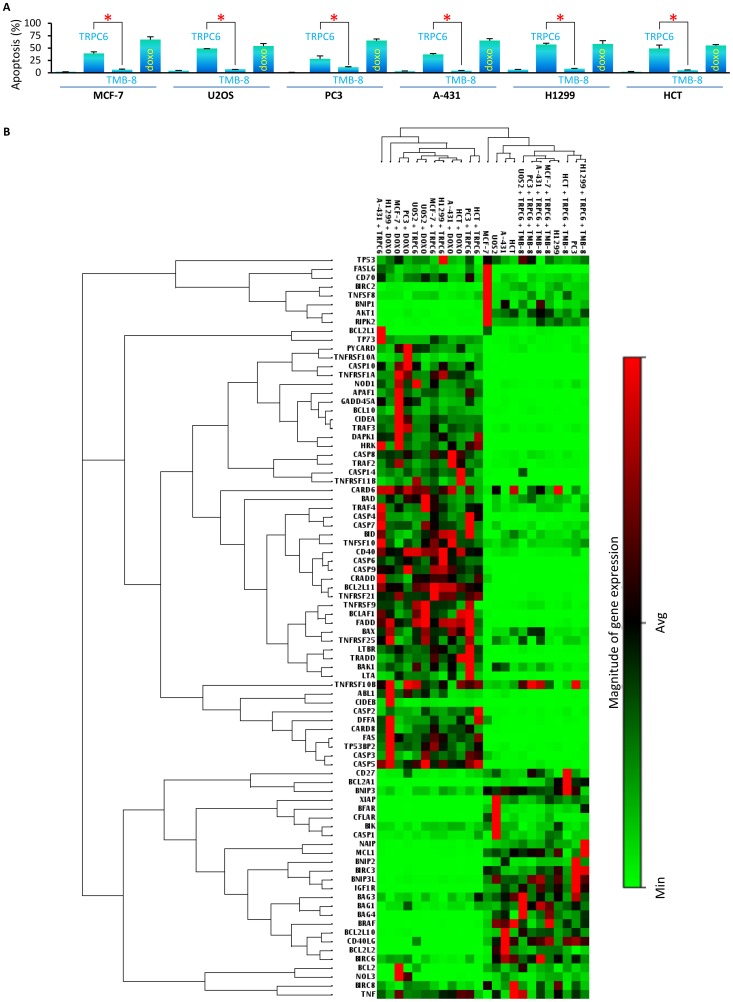
TRPC6 induces apoptosis and selectively upregulates apoptotic gene cluster in cancer cells. **A**) The effect of TRPC6 over-expression is observed upon cellular apoptosis in p53 wild-type cells (MCF-7, U2OS, HCT p53 (+/+)), p53 mutant cells (A-431, PC3) and p53 null cells (H1299) using annexin-V staining and the flow-cytometry. Significant increase in apoptotic fraction of TRPC6 over-expressing cells is seen irrespective of p53-gene status. Intracellular calcium quenching via TMB-8 abolishes TRPC6-mediated apoptosis; untreated cells serves as negative control, doxorubicin serves as positive control (* represents significant difference between TRPC6-treated cells and TRPC6-treated and TMB-8-treated cells; all p values <0.05, n = 7; S.D, Anova). (**B**) TRPC6 induces expression of apoptotic genes in cancer cells. PCR gene array for genes involved in regulation of cellular apoptosis is conducted in TRPC6 cDNA transfected p53 wild-type cells (MCF-7, U2OS, HCT p53 (+/+)), p53 mutant cells (A-431, PC3) and p53 null cells (H1299) cells. Over-expression of TRPC6 results in down-regulation of genes inhibiting apoptosis and up-regulation of pro-apoptotic genes (lane 1, 5, 7, 8, 11 & 12). Intracellular calcium quenching in TRPC6 over-expressing cell results in inhibition of apoptotic gens and increase in the expression of anti-apoptotic genes (lane 17, 18, 19, 20, 22 & 24). Untreated cells also serve as controls (lane 13, 14, 15, 16, 21 & 23); doxorubicin treatment serves as positive control which leads to upregulation of genes involved in apoptosis (lane 2, 3, 4, 6, 9 & 10) (n = 10).

We further analyzed the physiological relevance of this p53-dependent regulation of TRPC6 expression and the intracellular calcium levels. Since p53 is a key player in governing cellular apoptotic strategies in cancer cells. We hypothesized that p53-dependent expression of TRPC6 might be another cellular pathway through which p53 might induce apoptosis in cancer cells. Thus the effect of TRPC6 expression on cellular apoptosis was analyzed in p53 wild-type cells (MCF-7, U2OS, HCT p53 (+/+)), p53 mutant cells (A-43, PC3) and p53 null (H1299) cells. These cell lines were ectopically expressed with TRPC6 cDNA **(** **Figure 4a** **)** and the cellular apoptosis was compared with the control (un-transfected cells) cells using annexin-V staining and the flow cytometry. The results showed a significant increase in the apoptotic fraction of the TRPC6 overexpressing cancer cells, irrespective of the status of p53 gene. Next we observed the role of intracellular calcium in TRPC6-mediated apoptosis by quenching intracellular calcium release via TMB–8. The results showed that quenching of the intracellular calcium release via TMB-8 abolished TRPC6-mediated apoptosis in cancer cells. This data suggested that the TRPC6-dependent increase in the intracellular calcium release was crucial for apoptosis, thus the p53-dependent increase in the TRPC6 expression in cancer cells might be a strategy to induce apoptosis via calcium-mediated pathways. This might be another cellular pathway adopted by p53 to evoke the apoptotic response and function as the prime tumor suppressor molecule in cancer cells. This data establishes TRPC6 as a potential apoptotic protein which in future might sow potential to function as an anti-cancer gene-therapy molecule. In order to understand TRPC6-induced apoptosis in details we observed the activation and suppression of a key set of 84 genes (SA-Bio sciences, PAHS012) involved in both induction and inhibition of apoptosis. The mRNA expression analysis of these genes was conducted in p53 wild-type cells (MCF-7, U2OS, HCT p53 (+/+)), p53 mutant cells (A-43, PC3) and p53 null (H1299) cells **(** **Figure 4b** **)**. In the experimental set up these cells were overexpressed with the apoptotic TRPC6 cDNA. The results were consistent in all the cell lines and showed that TRPC6 over-expression resulted in the downregulation of the genes involved in inhibition of apoptosis and upregulated the genes involved in inducing cellular apoptosis **(** **Figure 4b** **(lane 1, 5, 7, 8, 11 & 12))**. Interestingly, upon ectopic expression of TRPC6 cDNA in cancer cells, where intracellular calcium was quenched through TMB-8 treatment, the gene expression pattern was altered and the expressions of the genes involved in cellular apoptosis was consistently low **(** **Figure 4b** **(lane 17, 18, 19, 20, 22 & 24))**. Treatment with doxorubicin, used as positive control, resulted in upregulation of genes involved in apoptosis **(** **Figure 4b** **(lane 2, 3, 4, 6, 9 & 10))**. Untreated cancer cells serve as controls **(** **Figure 4b** **(lane 13, 14, 15, 16, 21 & 23))**. This data suggests that TRPC6 induces apoptosis via increase of intracellular calcium levels and through this pathway it alters the expression of genes involved in apoptosis, suggesting that TRPC6 is a potential candidate for pro-apoptotic anti-cancer strategies. This novel molecular pathway through which p53 upon activation by GaQ_3_ transcriptionally regulates the expression of the pro-apoptotic TRPC6 calcium channel, which in turn inflicts calcium-mediated apoptosis is an interesting strategy adopted by p53 to function as a tumor suppressor. Further this pathway can be exploited to gain anti-cancer benefits in future.

## Discussion

In this research work we have shown that the intracellular calcium release is under the transcription control of the p53 tumor suppressor, via p53-dependent transcriptional regulation of TRPC6 gene. Genome wide analysis of p53 binding sites in the promoter regions of calcium channels showed presence of p53 RE in TRPC6 gene. We have shown that GaQ3 induces expression of TRPC6 only in cancer cells with wild-type p53 and p53 gene silencing reverses the GaQ3-induced increase in TRPC6 expression. This data also establishes that TRPC6 is a direct transcriptional target of p53 and p53 binds to its response element in the TRPC6 promoter. We have found the p53 binding site in the 0.6 Kb TRPC6 gene promoter which lies 400 base pairs upstream of the +1 transcription start site. The sudden rise in intracellular calcium release and calcium-mediated cellular apoptosis in GaQ_3_-treated cancer cells is due to p53-dependent transcriptional activation of TRPC6 gene. In this research work we have shown a direct connection between the p53 transcriptional ability and calcium signalling.

Understanding the relation between calcium signalling and p53 is important in cancer perspective since both these factors regulate cell-growth, differentiation, ageing, proliferation at the physiological, cellular and molecular level [12], [13]. In past a Ca^2+^-permeable TRPC channel has been shown to participate in a diverse array of cellular functions by regulating intracellular Ca^2+^ signaling [14]. TRPC6-mediated Ca^2+^ signaling activates cell-proliferation gene like calmodulin-dependent protein kinase and mitogen-activated protein kinase [15]. The role of TRPC6 in cancer growth and development is not clear; however multiple mechanisms are involved in TRPC6 channel activation and regulation in cancer cells. The physiological and cellular factors involved in cancer disease progression are also known to regulate the cellular TRPC6 expression [16]–[20]. The cellular factors like membrane receptor activation via TFN-α and cellular Ca^2+^ store depletion involved in cancer progression have been known to induce TRPC6 expression [16]–[20]. Recently role of an important signaling molecule ROS in TRPC6 activation is observed [18]. Since ROS is involved in both cancer progression and its regulation, thus it is important to identify the role of TRPC6 protein in the cancer disease development or regulation. Recent studies have shown that several pro-apoptotic factors, including members of the Bcl-2 family proteins and reactive oxygen species (ROS) regulate the Ca^2+^ sensitivity of both the Ca^2+^ release channels in the ER and mitochondria [21]. Further no data related to the relation between the tumor suppressor p53 and regulation of calcium signaling transcriptional regulation of TRPC6 gene in cancer cells is available. It is important to identify the relation between p53 and its control of the intracellular calcium release and the role played by TRPC6 gene in GaQ_3_-treated cancer cells. The p53 and TRPC6 induced since changes in the cytosolic concentrations of Ca^2+^ can induce signaling pathways that regulate a broad range of cellular events, including those important in tumorigenesis [21].

Further a new relation between the intracellular Ca^2+^ release and p53 was observed where Ca^2+^ release stabilized the binding of p53 to its transcriptional co-activator p300 and also stabilized the binding of p53-p300 transcriptional complex to the p53-DNA binding site on p53-minimal promoter [22]. In this research work we have elucidated the presence of a cellular cross-talk between p53 and calcium signalling, where calcium signalling activates p53 transcriptionally [6] and the active p53 increase the intracellular calcium release by transcriptionally regulating the promoter of TRPC6 gene. Thus calcium-dependent apoptosis might be mediated by p53 and calcium signalling might play a crucial role in p53-mediated apoptosis. Recently TRPC6 gene has shown crucial role in pathological cardiac hypertrophy and remodelling in response to stress [23], [24]. Deletion of TRPC6 prevents stress-induced exaggerated cardiac remodelling and overexpression of TRPC6 develop spontaneous cardiac hypertrophy in the mice model, suggesting towards a possible link between TRPC6 expression and cell-death and division, since cardiac remodelling involves both these processes. TRPC6 expression is also known to induce podocyte cytoskeletal remodelling [25], human keratinocyte differentiation [26], and hippocampal neuron differentiation [27]. An important role of TRPC6 has also been discovered in wound healing where a genome-wide screen identified TRPC6 important for myofibroblast transformation and TRPC6 gene-deleted mice showed impaired dermal and cardiac wound healing after injury. The previous reports suggest towards a link between TRPC6 expression and regulation of cell-division and cell-death related processes, in this report we are suggesting a strong role of TRPC6 as a p53-regulated pro-apoptotic protein in the cancer model. In conclusion, here we have elucidated a novel role of TRPC6 calcium channel to function as a p53 downstream effector protein which induces apoptosis by regulating cellular calcium levels in cancer cells. This pathway appears to be a strategy adopted by p53 protein to utilize calcium signalling as an effective pro-apoptotic anti-cancer means by transcriptionally regulating TRPC6 calcium channel, thus using TRPC6 as a possible mechanism to function as a tumor suppressor.

## Supporting Information

File S1 **Supplementary material and methods.**
(DOC)
