# Supplementary material for: p53 Increases Intra-Cellular Calcium Release by Transcriptional Regulation of Calcium Channel TRPC6 in GaQ3-Treated Cancer Cells
Source: PLoS One. 2013 Aug 16;8(8):e71016. doi: 10.1371/journal.pone.0071016 (PMC3745406; doi:10.1371/journal.pone.0071016)
Supplement: File S1 — Supplementary material and methods. (DOC) [file pone.0071016.s001.doc]

# p53 Increases Intra-cellular calcium release by transcriptional regulation of calcium channel TRPC6 in GaQ_3_-treated cancer cells

**Esha Madan1, ^, *, Rajan Gogna1, ^, *, £, Bernhard Keppler^2^, Uttam Pati^1, £^**

**^1^** Transcription and Human Biology Laboratory, School of Biotechnology, Jawaharlal Nehru University, New Delhi, Delhi, India. **^2^** Institute of Inorganic Chemistry, Vienna University, Vienna, Austria. **^** Present Address: Institute of Cell Biology, University of Bern, Bern, Switzerland. * Both authors contributed equally to this research work. **^£^** Corresponding authors.

***^£^ ADDRESS CORRESPONDENCE TO:***

***Uttam Pati****,* School of Biotechnology, Jawaharlal Nehru University, New Delhi, India, Email: [uttam@mail.jnu.ac.in](mailto:uttam@mail.jnu.ac.in), phone: +91-9312401811.

***Rajan Gogna***, School of Biotechnology, Jawaharlal Nehru University, New Delhi, India.

Present Address: Institute of Cell Biology, University of Bern, Bern, Switzerland, Email: [rajan.gogna@izb.unibe.ch](mailto:rajan.gogna@izb.unibe.ch), phone: +41-0791793735.

***MATERIAL & METHODS***

***Luciferase Assay:*** Cells were plated in 35 mm petri dishes the day before transfection so that they reached 60–80% confluence upon transfection. Reporter plasmids (1.0–1.5 mg/well) were transfected with effectene transfection reagent (Qiagen) as per the manufacturer’s instructions. After desired treatment period the cells were washed in cold PBS three times and lysed with 200ml of the lysis buffer by a freeze-thaw cycle, and lysates were collected by centrifugation at 14,000 rpm for 2 minutes in a bench top centrifuge. Twenty micro liter of supernatant was used for the assay of luciferase activity using a kit (promega) as per the manufacturer’s instruction.

***Real time PCR:*** Real time PCR was performed using the 7500 fast real-time system (applied biosystems) using taqMan probe (applied biosystems) (Lu *et al*., 2006). GAPDH served as an endogenous control to normalize expression. Each sample was analyzed in quadruplicate. Relative expression and standard error were calculated by the supplied fast 7500 real-time system software.

***Reverse transcriptase PCR:*** Cells were lysed in appropriate amount of trizol (1 ml trizol per well of a 6 well plate for cultured cells). Cells were repeatedly and vigorously pipetted. Cells were then kept at room temperature for 5-10 minutes, and then 200 ul of chloroform per 1 ml of trizol was added and mixed thoroughly. The cells were again left at room temperature for 10 mins. Cells were then centrifuged at 12,000 rpm at 4°C for 15 mins and the upper aqueous colorless layer was transferred to a fresh eppendorf tube. To this eppendorf tube 75ul LiCl (lithium chloride) followed by 1ml chilled EtOH (ethanol) were added and kept at -20°C for 2-3 hrs. The eppendorf tube was centrifuge at maximum speed (>=12000 rpm) for 15 mins at 4°C. The supernatant was discarded and 250ul of 70% EtOH was added and the tube was kept at room temperature for 2 mins. The tube was again centrifuged at 7500 rpm for 5 mins at 4°C, finally the supernatant was discarded and the pellet was re-suspended in RNA grade water till it was completely dissolved. Single strand c-DNA was synthesized for treatment with sense and anti-sense primers using revert aid TM h minus first strand cDNA synthesis (fermentas). The resulting cDNA was diluted 1:10 before proceeding with the PCR reaction. PCR was conducted in mastercycler gradient (brinkmann instruments inc.). Each PCR reaction used 50 µl cDNA, 2.5 U Taq polymerase (Eppendorf scientific inc.), 0.2 mM dNTPs and 0.5 µM primer. PCR products were resolved on 2% agarose gel containing 0.01% (v/v) ethidium bromide and visualized by UV illuminator. The size of the PCR amplicon was determined by comparison with 100-bp DNA ladder (Promega).

***RT-PCR Primers:***

β-Actin forward: 5’ ATGAAGTGTGACGTTGACATCCG 3’

β-Actin reverse: 5’ GCTTGCTGATCCACATCTGCTG 3’ p53

TRPC6 forward: 5’ TATTGAATTGCCGTGCATGTTAGCCCTAG 3’

TRPC6 reverse: 5’ GGCTATTACTAATAATTCGTACGTCAGTAAGATACGTAC 3’

***Western blotting:*** Whole-cell lysates were prepared using RIPA buffer (10 mM Tris-HCl [pH 7.4], 150 mM NaCl, 1% NP-40, 1 mM EDTA, 0.1% SDS, and 1 mM DTT). Cytoplasmic and nuclear protein fractions were prepared using digitonin. Proteins were resolved by 10% or 12% SDS-PAGE and transferred onto PVDF membranes (Invitrogen). Incubations with primary antibodies were followed by incubations with the appropriate secondary antibodies (amersham) and detection by ECL (Amersham). To quantify Western blotting signals, densitometry was performed using the software ImageJ.

***Electrophoretic mobility shift assay:*** The p53 EMSA were performed as described (Hainaut and Milner, 1993). Fifty nanograms of purified protein was mixed with 9 μl of binding buffer (HEPES pH 7.6 20 mM, NaCl 10 mM, MgCl2 1.5 mM, EDTA 0.2 mM, glycerol 20%, NP-40 0.1%, DTT 1 mM and PMSF 0.5 mM), 0.6 μl DTT, 2 μl salmon sperm, 1 μl BSA) and biotin-labeled probe. For super-shift experiments, 1 μl of the anti-p53 antibody 1801 (Oncogene Research Products, Cambridge, MA, USA) was added before incubation.

***Preparation of nuclear and cytoplasmic extracts:*** Cells were collected with a rubber policeman, and pelleted by centrifugation. 200µl of cytoplasmic extraction reagent (CER-I) (according to manufacturer’s instructions (#78833; ne-per tm nuclear and cytoplasmic extraction kit, Pierce inc.) was added per 20µl of cell volume, and the cell pellet was votexed for 15 sec. Cells were treated in presence of CER I for 10 mins, followed by treatment with 11µl of CER II and were further treated for 1 minute. Lysed cells were centrifuged at 13,000 rpm for 5 mins to pellet the intact nuclei. The supernatant containing the cytoplasmic fraction was carefully separated. The pelleted nuclei were re-suspended in 100µl of NER I, votexed for 15 sec and treated on ice for 45 mins with periodic votexing after every 10 mins. At this point the suspension was centrifuged at 13,000 rpm and the supernatant carrying the nuclear proteins were stored at -80 °C.

***Chromatin immunoprecipitation*:** Formaldehyde was added at a final concentration of 1% directly to cell culture media. Fixation proceeded at 22°C for 10 min and was stopped by the addition of glycine to a final concentration of 0.125 M. The cells were collected by centrifugation and rinsed in cold phosphate-buffered saline. The cell pellets were re-suspended in swelling buffer (10 mM potassium acetate, 15 mM magnesium acetate, 0.1 M Tris [pH 7.6], 0.5 mM phenylmethylsulfonyl fluoride, and 100 ng of leupeptin and aprotinin/ml), incubated on ice for 20 min, and then dounce homogenized. The nuclei were collected by microcentrifugation and then re-suspended in sonication buffer (1% sodium dodecyl sulfate, 10 mM EDTA, 50 mM Tris-HCl [pH 8.1], 0.5 mM phenylmethylsulfonyl fluoride, and 100 ng of leupeptin and aprotinin/ml) and incubated on ice for 10 min. Prior to sonication, 0.1 g of glass beads (212- to 300-µm diameter; Sigma) was added to each sample. The samples were sonicated on ice with an ultrasonics sonicator at setting 10 for six 20-s pulses to an average length of approximately 1,000 bp and then micro-centrifuged. The chromatin solution was pre-cleared with the addition of *Staphylococcus aureus* protein A-positive cells for 15 min at 4°C. Prior to use, the Staph A cells were blocked with 1 µg of sheared herring sperm DNA/µl and 1 µg of bovine serum albumin/µl for at least 4 h at 4°C. Pre-cleared chromatin from 10^7^ cells was incubated with 1 µg of affinity-purified rabbit polyclonal antibody or no antibody and rotated at 4°C for approximately 12 to 16 h. Prior to the first wash, 20% of the supernatant from the reaction with no primary antibody for each time point was saved as total input chromatin and was processed with the eluted immunoprecipitates beginning at the cross-link reversal step. Cross-links were reversed by the addition of NaCl to a final concentration of 200 mM, and RNA was removed by the addition of 10 µg of RNase A per sample followed by incubation at 65°C for 4 to 5 h. The samples were then precipitated at
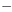
20°C overnight by the addition of 2.5 volumes of ethanol and then pelleted by micro-centrifugation. The samples were re-suspended in 100 µl of Tris-EDTA (pH 7.5), 25 µl of 5× proteinase K buffer (1.25% sodium dodecyl sulfate, 50 mM Tris [pH 7.5], and 25 mM EDTA), and 1.5 µl of proteinase K (Boehringer Mannheim) and incubated at 45°C for 2 h. Samples were extracted with phenol-chloroform-isoamyl alcohol (25:24:1) followed by extraction with chloroform-isoamyl alcohol and then precipitated with 1/10 volume of 3 M NaOAc (pH 5.3), 5 µg of glycogen, and 2.5 volumes of ethanol. The pellets were collected by micro-centrifugation, re-suspended in 30 µl of H_2_O, and analyzed by PCR. Forward primers were designed spanning the regions 101374658-101374678 and reverse primers were designed spanning the region 101374693-101374717 on Chr 11 respectively.

***REFERENCES***

Hainaut, P., and Milner, J. (1993). Redox modulation of p53 conformation and sequence-specific DNA binding in vitro. Cancer research *53*, 4469-4473.
